# Case report: Enamel renal syndrome: a case series from sub-Saharan Africa

**DOI:** 10.3389/froh.2023.1228760

**Published:** 2023-08-22

**Authors:** I. A. Roomaney, S. Kabbashi, K. Beshtawi, S. Moosa, M. Y. Chothia, M. Chetty

**Affiliations:** ^1^ Department of Craniofacial Biology, Pathology and Radiology, Faculty of Dentistry, University of Western Cape, Cape Town, South Africa; ^2^ Department of Dental Sciences, Faculty of Graduate Study, Arab American University, Jenin, Palestine; ^3^ Division of Molecular Biology and Human Genetics, Stellenbosch University Faculty of Medicine and Health Sciences, Cape Town, South Africa; ^4^ Medical Genetics, Tygerberg Hospital, Cape Town, South Africa; ^5^ Division of Nephrology, Department of Medicine, Faculty of Medicine and Health Sciences, Stellenbosch University and Tygerberg Hospital, Cape Town, South Africa

**Keywords:** enamel renal syndrome, fam20A, rare disease Africa, craniofacial manifestations, nephrocalcinosis

## Abstract

Enamel Renal Syndrome (ERS) (OMIM # 204690) is a rare genetic condition characterised by hypoplastic amelogenesis imperfecta, failed tooth eruption, intra-pulpal calcifications, gingival enlargement and occasionally nephrocalcinosis. In this case series, we report on four unrelated patients with a confirmed molecular diagnosis of ERS (*FAM20A* pathogenic variants) from Sub-Saharan Africa. The pathognomonic oral profile of ERS was mostly fulfilled in these patients, with the notable addition of an odontoma in one patient. The cases presented a spectrum of phenotypic severity both dentally and systemically. One patient presented with nephrocalcinosis and abnormal kidney function, one had reduced kidney size with normal kidney function, and two had no renal abnormalities. Patients presenting with the oral profile of ERS should receive a prompt referral to a nephrologist and a geneticist. They should receive long-term management from a multidisciplinary medical and dental team.

## Introduction

Enamel Renal Syndrome (ERS) (OMIM # 204690) is a rare genetic condition characterised by hypoplastic amelogenesis imperfecta (AI), failed tooth eruption, intra-pulpal calcifications, gingival enlargement and, in some patients, nephrocalcinosis (1). In 1972, MacGibbon described two cases with generalised enamel hypoplasia and renal dysfunction (2). Several studies have since described conditions with a similar phenotypic oral presentation, with and without renal involvement. These include AI with nephrocalcinosis, AI syndrome, AI with inter-radicular dentine dysplasia, AI with gingival fibromatosis (AIGFS), MacGibbon syndrome, and Lubinsky-MacGibbon syndrome (3, 4). In 2011, a pathogenic variant in the *FAM20A* gene (*FAMily with sequence similarity 20A*) was implicated (5). This discovery was later confirmed in 2012 by a large international consortium, which observed 25 cases with the oral phenotype and nephrocalcinosis (6). The absence of strict characterisation of ERS has led to patients' renal status being overlooked and has resulted in an underestimation of the actual disease prevalence (4).

Patients with ERS are likely to seek dental care first due to the retention of primary teeth and failure of permanent tooth eruption (7). A review by de la Dure-Molla and colleagues (2014) suggested a distinctive pathognomonic oral profile for ERS patients (4); however, atypical features are being more frequently reported. These include sensorineural hearing loss, hypertrichosis (8), hypodontia (9), and periodontal disease (1, 10–12). A recent study also identified two unrelated patients with pathogenic *FAM20A* variants, one presenting with early eruption of permanent teeth and another with the normal eruption of all 32 teeth (8). Similarly, the prevalence of nephrocalcinosis in patients with ERS is unknown. Nephrocalcinosis has been reported in several studies (1, 6, 9–14); however, others have failed to identify renal involvement (8, 15–18). The renal phenotype, typically silent during childhood, is characterised by reduced calcium, phosphate, and citrate excretion with subsequent nephrocalcinosis (4).

There is a paucity of genetically confirmed cases of ERS from Sub-Saharan Africa. Case reports by Van Heerden et al. (1990), Peters et al. (1992), and Feller et al. (2006) are some of the earliest publications describing patients with oral profiles resembling that of ERS in South Africa, albeit under different nomenclatures [“Rough hypoplastic AI with Follicular hyperplasia”, “Enamel dysplasia with odontogenic fibroma-like hamartomas”, and “AI associated with multiple impactions and odontogenic fibromas (WHO) type”] (19–21). This suggests that the prevalence of ERS in the region may be higher than previously considered. Here we present four genetically confirmed cases of ERS from Sub-Saharan Africa and highlight the importance of the diagnosis in patient management.

### Case description

This case series is a component of a project that was approved by the University of Western Cape (UWC) Biomedical Research Ethics Council (study number: BM21/7/16). The patients have provided written, informed consent for the publication of their data and images. The features of the patients found in this report are summarised in Table 1 and compared with the pathognomonic profile of ERS described by de la Dure-Molla et al. (13).

**Table 1:** Features of ERS in this case series compared to the pathognomonic oral profile to de la Dure-Molla *et al.* (4).

	Case 1	Case 2	Case 3	Case 4
*FAM20A* pathogenic variant	+	+	+	+
Generalised thin hypoplastic or absent enamel	+	+	+	+
Flat cusps of posterior teeth	Only 1 erupted posterior tooth- normal cusps	No erupted posterior teeth	+ Erupted deciduous teeth	+
Relative microdontia or spaced teeth	+	+	+	+
Delayed tooth eruption	+	+	+	+
Impacted posterior teeth with a hyperplastic follicle (hamartoma-like) and altered eruption pathway	+	+	+	+
Root dilaceration of impacted teeth	+	+	+	+ Severe dilaceration of the maxillary central incisors
Gingival fibromatosis	+	+	+	+ Including the palatal aspect of maxillary teeth
Gingival and dental follicle ectopic calcifications	To be confirmed with histology	To be confirmed with histology	To be confirmed with histology	To be confirmed with histology
Additional features				
Semi-lunar shaped central incisors	**-**	**-**	+ mildly on 21	- Central incisors unerupted
Crown resorption of non-erupted teeth	Unknown	Unknown	Unknown	Unknown
Anterior open bite	-	-	-	-
Root hypercementosis and inter-radicular dentine dysplasia	Requires histological assessment	Requires histological assessment	Requires histological assessment	Requires histological assessment
Supernumerary teeth	-	-	-	-
Other oral features in the presented cases	• Odontoma in the 3rd quadrant • Pigmentation on the gingiva and palate • Pneumatisation of the frontal sinuses • Thinning of anterior alveolar ridges	• Mild pigmentation of the gingiva • **Missing teeth** • Pneumatisation of the frontal sinuses • Thinning of anterior alveolar ridges	• Less gingival fibromatosis • Thickening in the maxillary antra • Enlarged inferior, left turbinate. • Thinning of the cortical outline of the condyles	• Mild pigmentation on the gingiva • Hypoplastic maxillary antra • Pneumatisation of the frontal sinuses • Enlarged inferior turbinates. • Thinning of the cortical outline of the condyles
Systemic features				
Nephrocalcinosis	-	-	-	**+**
Other renal features	None	Small kidney sizes with normal kidney function, hypocitraturia	None	Kidney dysfunction, hypocitraturia
Other systemic features	Iron-deficiency anaemia	None	None	Gastrointestinal and ophthalmological disturbances

+- present; - -absent

### Case 1

A 26-year-old Shona-speaking female from Harare, Zimbabwe, presented to the UWC Dental Faculty with the main complaint of retained primary teeth. She was the only child of non-consanguineous parents. Two half-brothers from the father were unaffected. There was no family history of any genetic conditions. The patient's medical history revealed iron deficiency anaemia. She had been to several dentists previously who could not assist with her dental complaints.

**Figure 1:**
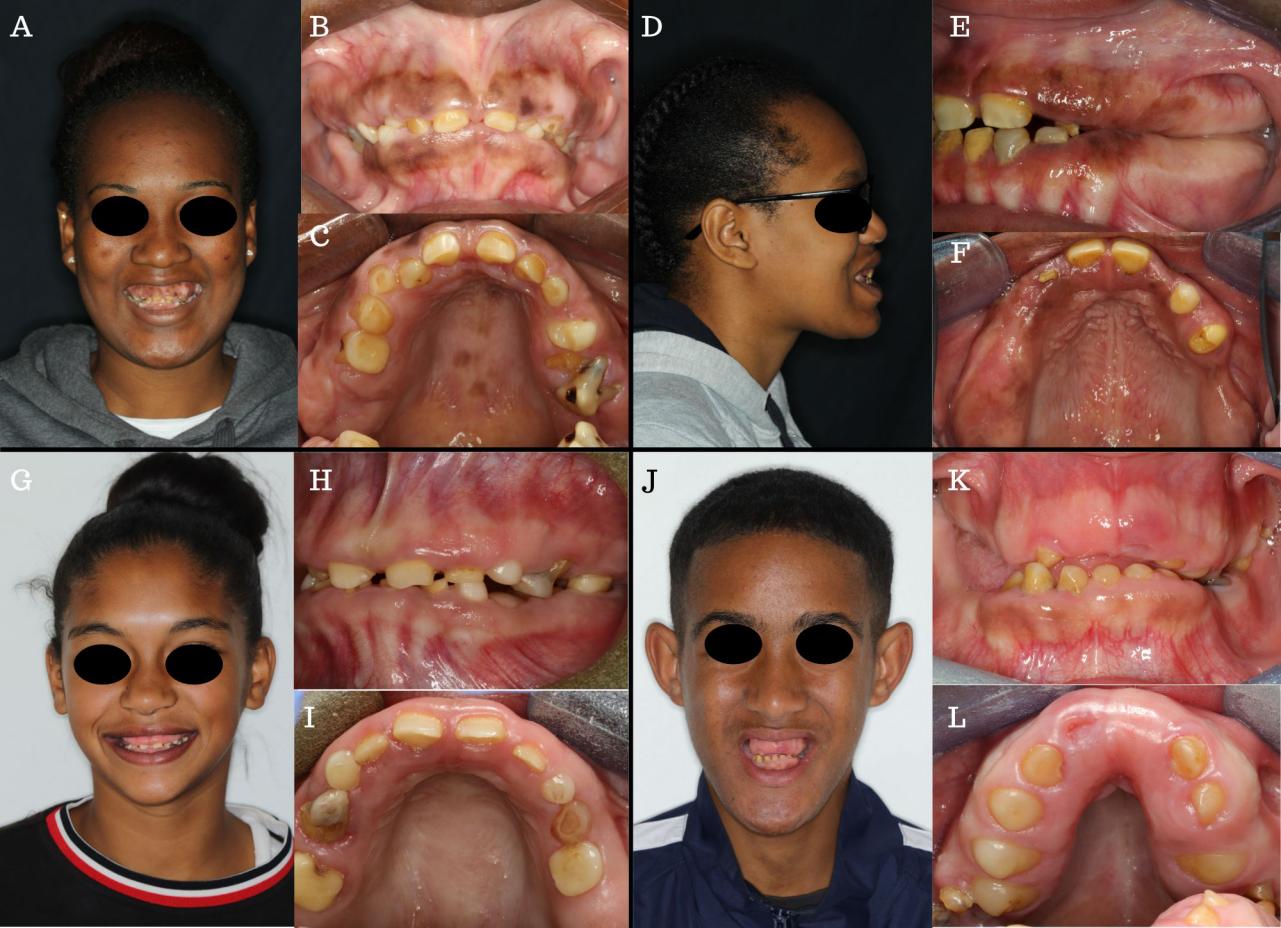
Clinical images of four patients with enamel renal syndrome*. Case 1*: (**A**): Profile showing no dysmorphic facial features. (**B,C**) Intra-oral images showing erupted primary and permanent teeth, fibrotic gingiva, and pigmentation on the attached and free-gingiva and the palatal mucosa. *Case 2*: (**D**) Lateral profile showing a mild Class III jaw relationship and no other dysmorphology. (**E,F**) Intra-oral images showing few erupted permanent teeth with variable enamel colourations, thick bone, and gingiva with no intermaxillary space posteriorly. Pigmentation is present on the attached gingiva*. Case 3*: (**G**): Profile view showing no dysmorphic facial features*.* (**H,I**): Intra-oral images showing mixed dentition, erupting premolar with no visible enamel and tooth wear. *Case 4*: (**J**) Profile picture showing vertical maxillary excess, thick arched brows, and protrusive ears. (**K,L**): Intraoral images showing severely thick, fibrotic gingiva and bulbous bone, severe tooth wear, and an absence of erupted maxillary incisors.

The extra-oral and intra-oral images are presented in Figures 1A–C. The patient had normal growth and no further dysmorphic features. The intra-oral examination revealed thick, fibrous gingiva and bulbous maxillary and mandibular alveolar ridges. Pigmentation was present on the attached gingiva and multiple round-shaped, macular, pigmentations were also present on the hard palate mucosa (Figure 1C). Due to the relative microdontia and wear, the permanent incisors, and premolars present intra-orally were almost indistinguishable from primary teeth. During occlusion, the posterior alveolar ridges were closely aligned, causing the patient to utilise them for mastication. The primary teeth showed marked attrition resulting in a smooth brown/amber surface. Only the incisal third of the maxillary anterior teeth were visible due to the hyperplastic gingiva and alveolar bone. The permanent teeth were yellow with a smooth, thin enamel surface.

**Figure 2:**
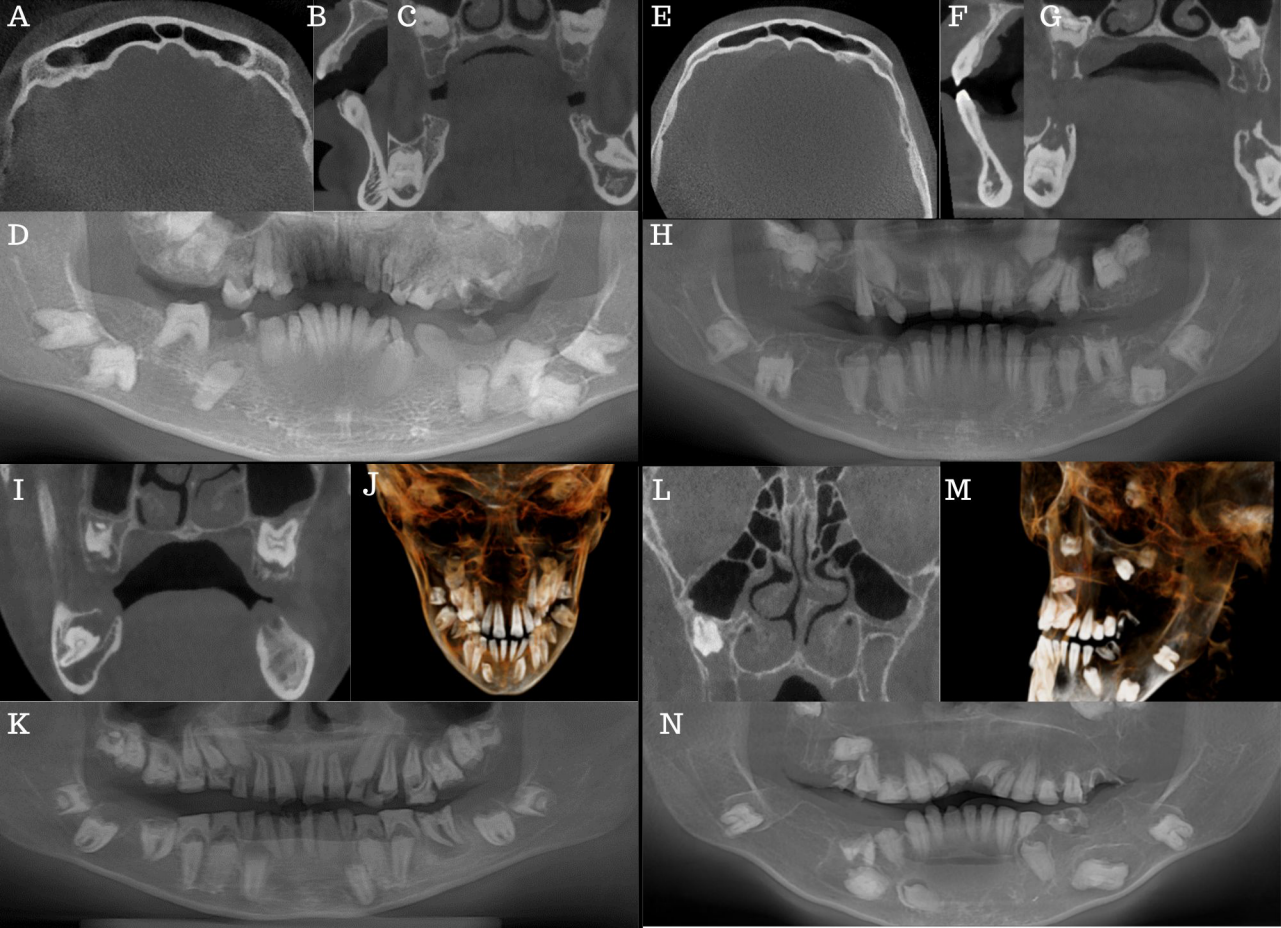
*Case 1* (**A–D**): (**A**) axial view showing frontal sinus pneumatisation. (**B**) Sagittal view showing severe thinning of the lower anterior alveolar ridge. (**C**) Coronal view showing several impactions, pulp stones, and enlarged dental follicles stars. (**D**) CBCT reformatted panorama. *Case 2* (**E–H**): (**E**) Axial view showing frontal sinus pneumatisation. (**F**) Sagittal view shows severe lower and upper anterior alveolar ridge thinning. (**G**): Coronal view showing several impactions, pulp stones and enlarged dental follicles stars. (**H**) CBCT reformatted panorama. Case 3 (**I–K**): (**I**) Coronal view showing several impactions, pulp stones, and enlarged dental follicles. (**J**) centre 3D model of the patient. (**K**) CBCT reformatted panorama. *Case 4* (**L–N**): (**L**) Coronal view showing hypoplastic maxillary antra. (**M**) Left sides of the 3D model of the patient. (**N**) CBCT reformatted panorama.

Radiologic examination (Figures 2A–D) showed multiple retained primary teeth (55, 53, 63, 64, 65, 75, 73 and 83), root remnants (26 and 85), infra-occlusion of the 15, and several impacted teeth (18, 17, 16, 13, 23, 24, 25, 27, 28, 38, 37, 36, 35, 33, 45, 47 and 48). Generalised, diffuse, intra-pulpal calcifications were noted, mainly in the coronal part of the primary and permanent teeth (Figure 2C). Teeth showed a delayed and aberrant eruption pattern. The molars had hypoplastic or absent enamel with flat cusps, and the roots were short and dilacerated. Enlarged dental follicles of teeth 18, 17, 24, 25, 27, 28, 38, 37, 33, 47, and 48 were noted. Pneumatisation of frontal sinuses (Figure 2A) and severe thinning of the upper and lower anterior alveolar ridges (Figure 2B) were present.

After the initial examination, the patient was referred to the geneticist and the nephrologist for consultation. The medical genetics evaluation yielded no additional abnormalities or dysmorphology, specifically in relation to structures that originate from the endoderm. A pathogenic variant of *FAM20A* was confirmed via exome sequencing. Laboratory results revealed normal kidney function and nephrocalcinosis was absent on ultrasound examination.

### Case 2

A 20-year-old Tsonga-speaking, female patient from Mpumalanga, South Africa, presented to the UWC Dental Faculty with the main complaint of difficulty eating due to a lack of teeth. She was the only child of non-consanguineous parents and had two unaffected half-siblings from the father. There was no significant family or medical history.

Extra-oral examination (Figures 1D–F) revealed a skeletal Class III jaw relationship with a protrusive mandible (Figure 1D). Her only erupted teeth were the 14, 11, 21, 22, 24,25 and the 42–32. The enamel showed inconsistent thickness, with some teeth appearing more yellow and others having a translucent, grey appearance (Figure 1E). The erupted 14 showed a complete absence of enamel (Figure 1F). Physiological pigmentation was visible on the attached gingiva and the alveolar ridge mucosa. The gingiva was thick and fibrous and showed no evidence of inflammatory changes. The ridges were thick and bulbous, particularly in the edentulous saddle region of the second quadrant (Figure 1E).

Radiologic examination (Figures 2E–H) showed missing teeth (16, 12, 26 and 46), a retained primary 53, and several tooth impactions (18, 17, 15, 13, 23, 27, 28, 35, 34, 38, 37, 44, 45, 47 and 48). Teeth 24,25, and 43 were not in occlusion. Intra-pulpal calcifications, delayed tooth eruption, hypoplastic enamel, and misshapen teeth with short, dilacerated roots were present (Figures 2G,H). Enlarged dental follicles of teeth 18, 17, 15, 13, 23, 27, 28, 38, 37, 35, 44, 45, 47 and 48 were noted. The frontal sinuses were pneumatised (Figure 2E), and severe thinning of the lower and upper anterior alveolar ridge was also noted (Figure 2F). The cortical outline of the right and left condyles was thin.

The medical genetic assessment found no additional dysmorphic features. A pathogenic variant of *FAM20A* was identified via exome sequencing. Although kidney sizes were small on ultrasound examination (left kidney: 8.5 cm and right kidney: 8.1 cm), no nephrocalcinosis was noted, and laboratory blood results revealed normal kidney function. A 24-hour urine collection revealed hypocitraturia of 0.2 mmol/day (reference: low urinary citrate <1.67 mmol/d) with normal urinary calcium-to-creatinine ratio of 0.35 mmol/mmol (reference range: 0.03–0.69 mmol/mmol) and urinary phosphate-to-creatinine ratio of 2.96 mmol/mmol (reference range: 0.13–6.47 mmol/mmol).

### Case 3

A 14-year-old female of mixed ancestry from Cape Town, South Africa, presented with the main complaint of pain from a carious 36. She was the youngest of three daughters from non-consanguineous parents and the only affected family member. There was no significant medical or family history.

On extra-oral examination (Figures 1G–I), no abnormalities were detected. She had a borderline Class III skeletal profile and clinically insignificant facial asymmetry (Figure 1G). Intra-orally, the patient was in the mixed dentition phase with several retained primary teeth. The primary teeth were worn and very smooth, with the enamel clearly visible (Figure 1H). The erupted permanent teeth showed a smooth enamel surface and wear of the incisal edges. The erupted 36 was broken down at the sub-gingival level and decayed. No enamel was visible on the erupting 24 (Figure 1I). Fibrous gingiva was present; however, in this instance, the gingiva did not appear as thick, and the alveolar ridge was not as bulbous as in the other cases. No physiological pigmentation was visible.

Radiologic examination (Figures 2I–K) revealed mild mucosal thickening in the maxillary antra and an enlarged inferior, left turbinate (Figure 2I). Thinning of the cortical outline of the right and left condyles were noted. A well-defined, corticated low density was present periapically to the 36 (Figure 2K). Intra-pulpal calcifications, delayed tooth eruption, hypoplastic enamel, and misshapen teeth with short, dilacerated roots were present (Figure 2K). The tooth follicles surrounding the unerupted teeth exhibited enlargement, albeit not as significantly as observed in the other cases.

The medical genetic assessment identified no additional dysmorphic features and a pathogenic variant of *FAM20A* was identified via exome sequencing. Laboratory blood results revealed normal kidney function, and no nephrocalcinosis was noted on ultrasound examination.

### Case 4

A 16-year-old male of mixed ancestry from Cape Town, South Africa, presented to the dental faculty with pain from a grossly carious 46. He was the only child of non-consanguineous parents and had no significant family history. His medical history revealed gastrointestinal and ophthalmological problems, which were being investigated and managed at the time. He reported progressive blurring of his vision with associated itching and dryness of the eyes. He suffered from recurring episodes of diarrhoea, abdominal cramps, and constipation. He also suffered from allergic rhinitis. He showed signs of mouth-breathing.

He presented with a vertical maxillary excess (Figures 1J–L) and an excessively gummy smile. He had a Class III malocclusion with a protrusive mandible and prominent chin. His ears were prominent, and he had thick, arched eyebrows (Figure 1J). Both his alveolar ridges were bulbous, with his maxillary ridge being significantly more affected, particularly on the palatal aspect where the ridges “crowded” the palate. This created the appearance of a very narrow, deep palate (Figure 1L). The gingiva was pale, thick, and smooth. Mild physiological pigmentation was visible on the attached gingiva of the mandibular ridge. The maxillary central incisors were unerupted. The teeth visible in the mouth were severely worn, microdontic, and spaced. The mandibular incisors were barrel-shaped, and the 36, the only permanent molar eruption, was also grossly carious. The erupted teeth had smooth enamel surfaces.

Hypoplastic maxillary antra and pneumatised frontal sinuses were present radiologically (Figure 2L). The inferior left and right turbinates were enlarged. Thinning of the cortical outline of the right and left condyles was noted. The 36 was extracted due to caries. Generalised foreshortening of teeth roots (particularly teeth 14, 15, 22, 25 and 34) was noted. The roots of 11 and 21 were severely dilacerated. The follicles around the mandibular molar teeth were enlarged and irregularly shaped.

The medical genetic assessment found no other dysmorphology, and a pathogenic variant of *FAM20A* was identified via exome sequencing. Laboratory blood results revealed mild kidney dysfunction with a serum creatinine concentration of 90 µmol/l [estimated glomerular filtration rate (modified Schwartz formula) of 67 ml/min/1.73 m^2^]. A 24-hour urine collection demonstrated hypocitraturia of 0.8 mmol/day, normal urinary calcium-to-creatinine ratio of 0.08 mmol/mmol, and normal urinary phosphate-to-creatinine ratio of 1.41 mmol/mmol. Nephrocalcinosis was present on ultrasound examination.

## Discussion

ERS is a rare genetic condition which often results in a pathognomonic oral phenotype and sometimes has severe systemic effects. This case series presents four unrelated patients with ERS from Sub-Saharan Africa. These cases were identified by dental personnel with a high clinical index of suspicion for the diagnosis. They were referred to the medical geneticist and nephrologist for further assessment and testing. This highlights the importance of recognising the oral profile, as the dentist is often the patient's first contact with the healthcare system.

Although the pathognomonic oral profile by de la Dure Molla et al. (4) helps identify those potentially having ERS; it is important to note that not all features are required for diagnosis. The cases presented a spectrum of phenotypic severity. The anterior teeth and primary teeth clinically appeared to have enamel of variable thickness; however, the unerupted molars and premolars had very little to complete absence of enamel. In some instances, it appeared that the crowns of the teeth did not completely develop. Whether this is due to crown resorption, as mentioned in prior studies (4), is debatable and would require long-term monitoring. Molars also tended to be impacted more frequently than incisors, with only one of our cases (Case 4) having impacted incisors. In Cases 1 and 4, molars were more severely embedded and unfavourably angulated compared to Cases 2 and 3.

The cases described had several features not previously or infrequently reported to be associated with ERS. Cases 3 and 4 had thinning of the cortical outline of the condyles and enlargement of the turbinates. Cases 1, 2, and 4 had pneumatisation of the frontal sinuses. Case 1 also had an odontoma, a novel finding associated with ERS. Additionally, gingival pigmentation was noted in three patients. It is unclear whether the pigmentation is a physiological characteristic related to ethnicity or a feature specific to ERS. Although the regular-bordered, round-shaped, macular pigmentation on Case 1's palatal mucosa is not typical of physiological pigmentation. Further genotype-phenotype association studies and standardisation of reporting are required to elucidate whether the phenotypic diversity can be attributed to the *FAM20A* variation or other factors. A genotype-phenotype correlation study will be published in the future.

Although the oral profile of ERS is quite striking, the clinician needs to exclude conditions with overlapping characteristics. Non-syndromic amelogenesis imperfecta, for instance, can manifest with enamel defects, ranging from chalky-white to rough, yellow, and brown appearances due to quantitative or qualitative enamel defects. Chronic renal disease may also result In enamel hypoplasia and drug-induced gingival enlargement. (22) However, neither of these conditions are associated with the enlarged dental follicles and displaced unerupted teeth with which ERS is associated. In comparison, gingival enlargement linked to chronic kidney disease usually appears more inflamed, with enlarged interdental papillae (22), whereas ERS-associated gingiva generally lacks the signs of inflammation. ERS can also be confused with specific syndromes, particularly Jalili, Raine, and tricho-dento-osseous syndromes (4). Jalili syndrome presents with rod-cone dystrophy and amelogenesis imperfecta (23). There have been no reports of ERS associated with rod-cone dystrophy. Raine syndrome, caused by a pathogenic variant of *FAM20C* (a paralogue of *FAM20A* and *FAM20B*), shares a similar oral phenotype to ERS, including ectopic mineralizations of the pulp and gingiva. However, extraoral features such as choanal atresia, midface hypoplasia, neurologic and orthopaedic problems, and hypophosphatemic rickets distinguish Raine syndrome from ERS clinically (24–26). Tricho-deno-osseous syndrome is associated with AI and taurodontism, but nail defects, bone sclerosis, and hair described as curly or kinky are defining characteristics (27). Ultimately, genetic testing to identify the *FAM20A* pathogenic variant is the gold-standard method of diagnosing ERS.

Renal findings in ERS patients have been variable. A systematic review reported that 53% of patients who had laboratory evaluation had metabolic abnormalities (28). These included hypocalciuria (11.6%), raised serum creatinine concentrations (7.2%), hypocitraturia and hypophosphaturia (5.8%). The mechanisms underlying nephrocalcinosis are yet to be determined; however, studies in murine models suggest that the *FAM20A* gene mutation may cause altered phosphorylation of proteins in renal tubular cells predisposing to nephrocalcinosis (29). Two of our four patients had renal involvement. Case 2, a 20-year-old female, presented with kidneys which were smaller in size and hypocitraturia. Case 4, a 16-year-old male, displayed nephrocalcinosis with associated kidney dysfunction. There is uncertainty about whether nephrocalcinosis develops with increasing age since some reports have identified nephrocalcinosis in very young patients (10, 14, 30–33), including patients as young as six years old (14, 30). In 2012, Jaureguiberry et al. (6) investigated 25 patients from 16 families with the ERS oral profile and nephrocalcinosis and speculated that all individuals with biallelic *FAM20A* mutations would eventually show nephrocalcinosis; however, several authors failed to identify any renal involvement in patients presenting with the typical oral profile of ERS (8, 15, 17, 18, 34). There has also been no observed correlation between sex and the severity of the phenotype, with both males and females showing equivalent case numbers (28), but there has been no comparison of severity. Only larger cohorts and long-term follow-ups will clarify the natural history of the systemic manifestations of ERS.

In patients with ERS, tooth structures are compromised to the extent that crown preparation and bonding are unlikely to succeed. Hall et al. (35) attempted surgical exposure and restorations using composite material, and ultimately pulp necrosis ensued, necessitating endodontic treatment. Surgical exposure and orthodontic alignment of teeth have a highly questionable prognosis due to compromised periodontal ligaments and severely ectopically positioned teeth. Nitayavardhana et al. (8) advised that embedded teeth be removed due to the progressive embedding of teeth with age and the development of dental infection. Mauprivez et al. (36) were the only authors to detail the dental rehabilitation of a patient with ERS. Their treatment plan consisted of surgical removal of all non-viable teeth and alveolar bone recontouring to create space for a removable complete maxillary denture and an implant-supported mandibular denture. All our cases have undergone surgical removal of embedded teeth interfering with prosthesis placement, recontouring of the alveolar ridge and will have implant-supported dentures constructed. The dental management will be reported later. Due to limited resources, the dental treatment has been protracted; however, the patients have remained motivated to continue with treatment. Individualised dental management and continuation of care over the life course is required to determine which dental treatment modalities are most successful and feasible.

Currently, no guidelines are available for managing patients with ERS. Our patients consulted private general dental practices on several occasions before attending our clinic. They were unable to receive adequate care due to the complexity of their required management and financial constraints, resulting in frustration and reduced quality of life. When asked about their perspective of their management journey, one patient responded: “*It's been a long journey. At first, l went for several consultations before anyone knew how to manage me. It took about three years for the hospital to finally start with treatment. I was excited for the treatment because l waited my entire life to get it done, and I was told it would take two years for me to get the results because l was going to get implants done. Unfortunately, due to doctors being changed, it will take more years, and it's not easy seeing specialists; it takes months or even a year before you get an appointment with them. My first surgery was done last year around October, and it was successful; l recovered very quickly. Also, due to my many scheduled appointments, and the distance I live from the hospital, it has cost me a lot of money. Every visit, I spend ZAR350,00–ZAR400,00 (USD15,00–USD20,00) on transport which takes a toll on me since I don’t earn that much. So, l hope in the future, the specialists and the hospital will come to a final decision which will not consume much time and more visits to the hospital*.” This statement highlights the challenges encountered by patients being treated in low-resourced settings. With improved knowledge of this condition, more streamlined management approaches can be achieved.

The sequence of care received by our patients is presented in Table 2. Patients suspected of having ERS require a full physical examination and should be referred to a geneticist where possible. Although the presence of nephrocalcinosis appears to be variable, all patients presenting with ERS require renal examination and long-term monitoring of renal state to allow for earlier detection and decrease sequelae associated with deteriorating renal function. Increased medical-dental collaboration will lead to better characterisation of this condition and allow for more effective and efficient management of those affected.

**Table 2:** Patient management according to CARE guidelines.

1. Initial patient assessment	Presenting symptoms	• Pain from caries • Retained primary teeth. • Non-eruption/ eruption of few permanent teeth • Difficulty eating • Poor dental aesthetics
	Patient history	• Medical • Familial • Dental
	Physical examination	• Malformed, spaced teeth • Delayed dental development. • Thick gingiva and bone around teeth • Amelogenesis imperfecta • Retained primary teeth, unerupted permanent teeth. • Tooth wear • No or subtle facial dysmorphology
	Radiologic examination	• Variable enamel thickness • Unerupted and malformed teeth, particularly molars • Pulp stones • Enlarged dental follicles around unerupted/ impacted teeth. • Bone and gingiva overgrowth • Dilaceration and shortened root • Sinus pneumatisation • Enlargement of the inferior turbinates • Thinning of the cortical outline of the condyles
	Differential diagnosis	• Enamel Renal Syndrome • Non-syndromic amelogenesis imperfecta • Syndromic amelogenesis imperfecta (Jalili syndrome, raine syndrome, epidermolysis bullosa, tricho-dento-osseous syndrome) • Renal disease
2. Referral	Dental	• General dentist • Paediatric dentist • Maxillofacial oral surgeon • Prosthodontist • Orthodontist
	Human Genetics	• Clinical examination • Genetic testing (gene-specific, gene panel for AI, exon sequencing, whole genome sequencing) • Genetic counselling
	Nephrological	• Clinical Examination • Blood tests for kidney function (serum creatinine, estimated glomerular filtration rate) • Spot urine analysis (protein-to-creatinine ratio) • 24-hour urine analysis (sodium, potassium, calcium, magnesium, uric acid, citrate, pH, phosphate, oxalate) • Renal ultrasound/ abdominal radiograph
3. Management	Dental	• Paediatric management: preservation of primary teeth • Removal of teeth and or bone impeding prosthetic management • Prosthetic rehabilitation • Life-long follow-up
	Nephrological	• Regular monitoring of kidney function

## Data Availability

The original contributions presented in the study are included in the article/Supplementary Material, further inquiries can be directed to the corresponding author.
